# Refractory Vasospastic Angina: When Typical Medications Don't Work

**DOI:** 10.7759/cureus.4134

**Published:** 2019-02-25

**Authors:** Varun Tandon, Christian M Mosebach, Manish Kumar, Saurabh Joshi

**Affiliations:** 1 Internal Medicine, University of Connecticut, Farmington, USA; 2 Cardiology, University of Connecticut, Farmington, USA

**Keywords:** refractory vasospastic angina, acute coronary syndrome, nstemi, stemi, vasospastic angina, prinzmetal angina

## Abstract

Vasospastic angina (VSA) is defined as spasm of the coronaries leading to transient constriction and eventual myocardial ischemia. VSA is treated typically with calcium-channel blockers (CCBs) and nitrates. However, there are times when the vasospasm is refractory to typical medications. When this occurs, unconventional treatment modalities may be employed for symptomatic relief. We present a case of a 48-year-old-male with a history of inferior ST-elevation myocardial infarction (STEMI) status post percutaneous coronary intervention (PCI) with drug-eluting stent (DES) to the distal right coronary artery (RCA), who presented with recurrent angina. The pain was described as pressure-like, substernal, radiating to both arms, and similar to his previous STEMI presentation. On presentation to the emergency room, he had an elevated serum troponin with no electrocardiogram (EKG) changes. He was taken to the cath lab where it was found that he revealed severe focal stenosis just proximal to the previously placed stent. Immediately after guidewire passage into the RCA, acute vasospasm developed, resulting in diffuse, severe stenosis, extending over previously normal segments to the proximal RCA, resolving with intracoronary nicardipine and nitroglycerin, including the initial focal stenosis. The patient was diagnosed with VSA. Unfortunately, despite optimal medical therapy, he developed refractory VSA, requiring the use of unconventional treatment methods. Our patient presented with a lesser-known phenomenon called refractory VSA, where intermittent vasospasm continues despite being on a combination of two medications. Treatment for VSA is well-documented, however, little data is available for refractory VSA.

## Introduction

Vasospastic angina (VSA) occurs when there is spasm of the coronaries, transiently leading to constriction and eventual myocardial ischemia. VSA is treated typically with calcium-channel blockers (CCBs) and nitrates [1]. However, there are times when the vasospasm is refractory to typical medications. When this occurs, unconventional treatment modalities may be employed for symptomatic relief. We present a case of refractory VSA, which required unconventional treatment for symptom control.

## Case presentation

**Figure 1:**
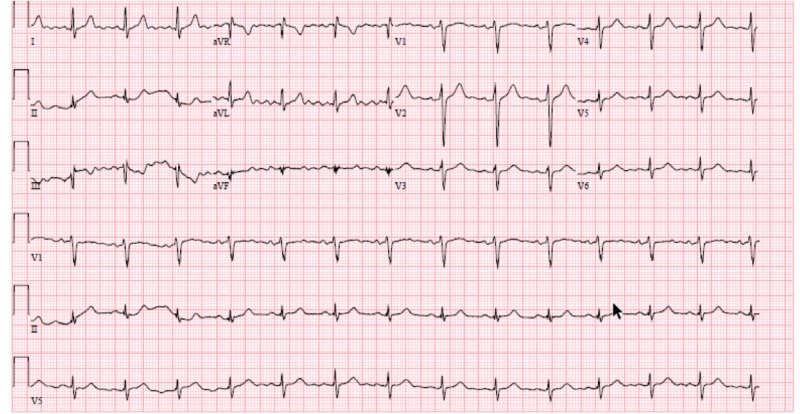
Electrocardiogram on presentation to the emergency room Normal sinus rhythm of 90 beats/min with normal axis and intervals. There is poor R-wave progression but no signs of acute ST-changes. There are old T-wave inversions in lead III.

**Figure 2:**
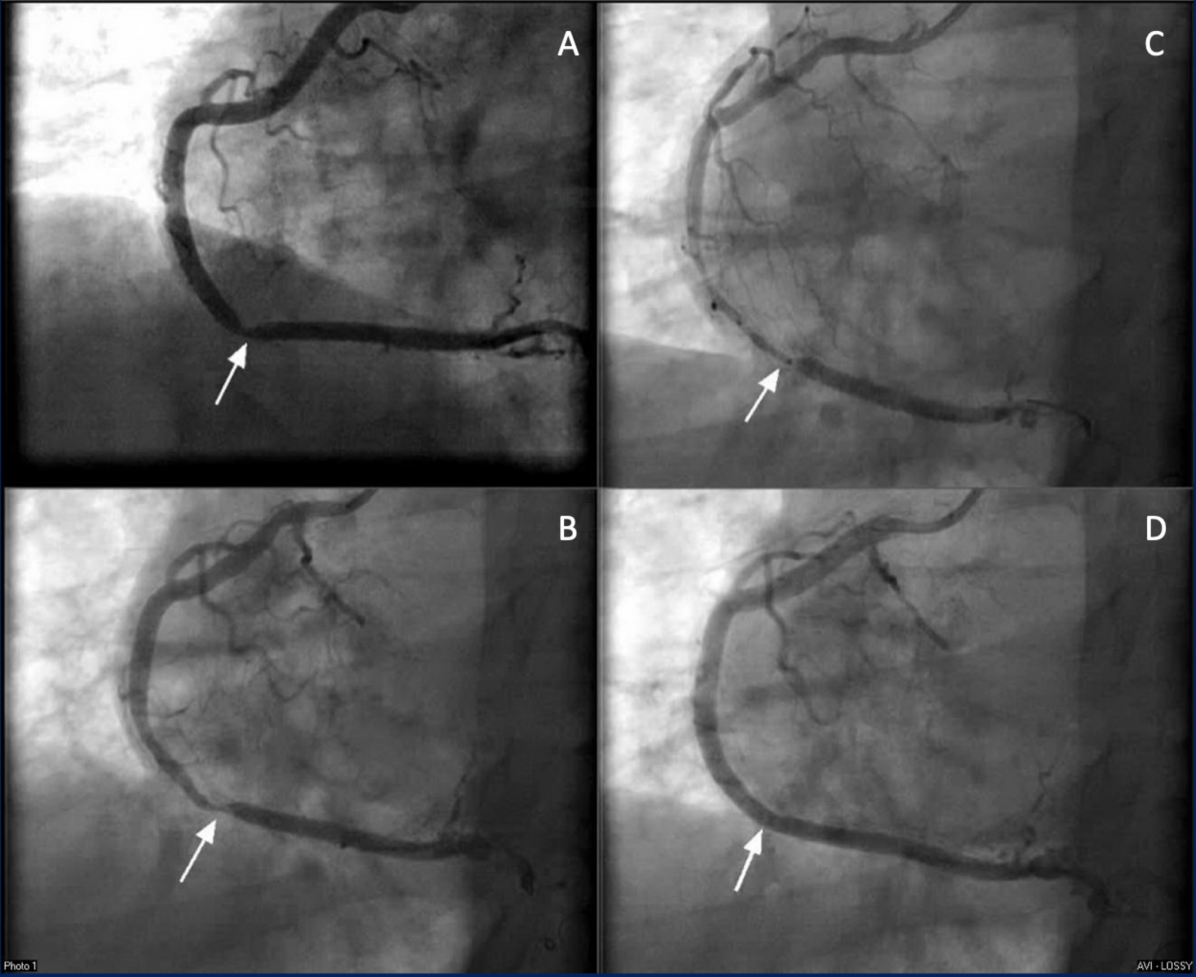
Left heart catheterization demonstrating RCA from LAO 30 A: Focal stenosis of 90% in the distal RCA, which was determined to be a result of vasospasm. B: Vasospasm in the distal RCA with guidewire introduction. C: Diffuse vasospasm with guidewire in place, expanding from the distal to the proximal RCA. D: Vasospasm resolved with intracoronary nitroglycerin and intracoronary nicardipine. RCA - Right Coronary Artery; LAO - Left Anterior Oblique

A 48-year-old-male with a history of inferior ST-elevation myocardial infarction (STEMI) status post percutaneous coronary intervention (PCI) with drug-eluting stent (DES) to the distal right coronary artery (RCA) eight months prior, presented with recurrent angina, described as pressure-like, substernal, radiating to both arms, and similar to his previous STEMI presentation. His angina occurred at rest and was alleviated with sublingual nitroglycerin. The patient was compliant with guideline-directed medical therapy with dual antiplatelet therapy (DAPT), statin, and beta-blocker (BB). His family history did not have any history of premature coronary artery disease or of sudden cardiac death. He never smoked and rarely consumed alcohol. His vitals on presentation to the emergency room were: blood pressure (BP) 146/82 mmHg; heart rate (HR) 88/min; respiratory rate (RR) 16/min; afebrile; and oxygen saturation of 98% on room air. His physical exam, including cardiac and pulmonary exams, were unremarkable. His electrocardiogram (EKG) demonstrated signs of prior inferior infarct with no acute signs of ischemia or ST-changes (Figure 1). Serum troponin was initially 0.37 ng/L (normal <0.05 ng/L) and subsequently peaked at 1.93 ng/L. The patient was diagnosed with non-STEMI. A heparin infusion was started per acute coronary syndrome (ACS) protocol. Given the diagnosis of non-STEMI, left heart catheterization was performed, revealing severe focal stenosis just proximal to the previously placed stent. A decision to proceed with PCI was made. Immediately after guidewire passage into the RCA, acute spasm developed, resulting in diffuse, severe stenosis, extending over previously normal segments to the proximal RCA. This completely resolved with intracoronary nicardipine and nitroglycerin, including the initial focal stenosis (Figure 2). The patient was diagnosed with vasospastic angina (VSA). He was continued on DAPT, BB, and statin with the addition of the non-dihydropyridine calcium channel blocker (CCB), verapamil. Despite this, the patient continued to experience intermittent angina and verapamil was increased to the maximum dose. An oral long-acting nitrate was additionally added but quickly discontinued due to intolerable headaches. Various second CCBs were added, including a dihydropyridine CCB, but intermittent angina continued. At this point, the patient was diagnosed with refractory VSA. Clonidine (alpha-2-agonist) was also tried, with no benefit. Eventually, a nitroglycerin patch was added with reduced headaches and a modest decrease in the frequency of angina episodes.

## Discussion

Angina pectoris was first described in 1768 by William Heberden when he presented the now classic symptoms of chest pain; occurring with effort/exertion, no EKG changes, and resolution with rest to the Royal College of Physicians [2]. In 1959, a variant form of angina pectoris, “Prinzmetal angina” was then described by Dr. Myron Prinzmetal with the symptoms of chest pain at rest, ST-elevation, and spontaneous resolution or resolution with the use of sublingual nitroglycerin [3]. With the advent of coronary angiography, the suspicion of coronary spasm as the cause of Prinzmetal’s angina was confirmed. Subsequently, it was found that spasm is not always associated with ST elevation. Therefore, the term “vasospastic angina” was coined.

**Figure 3:**
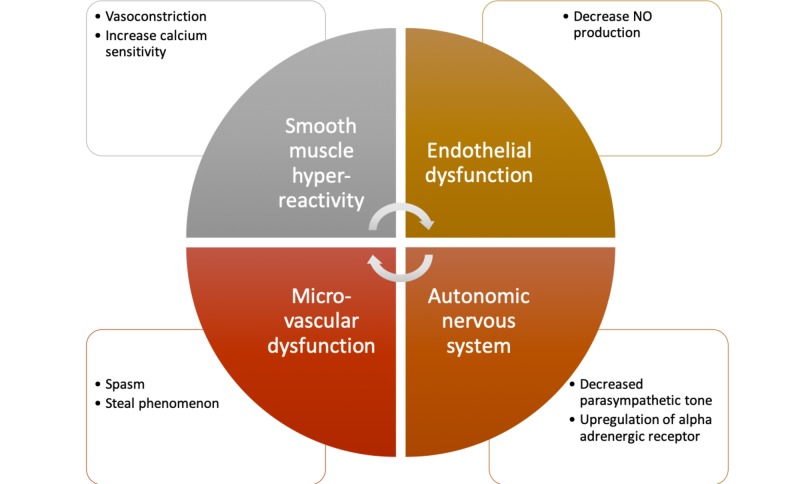
Vasospastic angina pathophysiology As smooth muscle hyperreactivity occurs, a cycle is initiated leading to spasm of the vessel and ischemia. If this spasm does not subside, infarction, arrhythmia, or even sudden cardiac death may occur.

In terms of pathophysiology, it is believed that there is significant overlap between atherosclerotic CAD and VSA. More than half of the patients with atherosclerotic CAD may develop vasospastic angina and more than half vasospastic angina patients have atherosclerotic CAD [4]. The inciting factor, whether a normal or a diseased vessel, is smooth muscle hyperreactivity. This subsequently leads to a focal or diffuse spasm, resulting in a high-grade obstruction. This obstruction ultimately leads to transient ischemia and anginal pain. If this persists, myocardial infarction, arrhythmia, or even sudden cardiac death may occur (Figure 3) [5].

Smooth muscle hyperreactivity can occur due to a variety of factors but, overall, is either due to vasoconstriction or an increase in calcium sensitivity of the vascular myosin light chains. Acetylcholine, serotonin, histamine, noradrenaline, and dopamine have all been shown to provoke vasospasms. The non-receptor pathway inhibitors of smooth muscle contractile mechanisms, such as that of nitrates and CCB, can inhibit spasms [6]. Smoking has been noted to be a major risk factor for coronary spasms whereas hypertension, hyperlipidemia, and diabetes mellitus, are not found to be large risk factors [7]. Other risk factors for vasospasm include, but are not limited to, hyperventilation, cold, exercise, magnesium deficiency, drugs (cocaine, marijuana, alcohol, ephedrine, amphetamine, triptans), and left heart catheterization (guidewire, balloon dilatation) [8-9].

Diagnosis can be made via left heart catheterization with provocation testing with, typically, acetylcholine or with ergonovine. It should ideally be done at centers that have high levels of expertise in the procedure. Overall, it is deemed a safe procedure, however, it can result in sustained spasm, serious arrhythmias, and even death, though very infrequently. If this approach is to be done, it is preferable to have a wash-out period for both nitrates and CCBs. Also, a temporary pacemaker should be placed in the right ventricle prior to provocation [8-9]. For acetylcholine administration, it is given as 20, 50, and 100 mcg intracoronary over 20 seconds and after one-minute angiogram is performed. This is then followed by nitrate administration and angiogram when maximally dilated. For ergonovine, it is administered as 20-60 mcg intracoronary over two to five minutes, and after one minute, an angiogram is performed [8-9].

**Figure 4:**
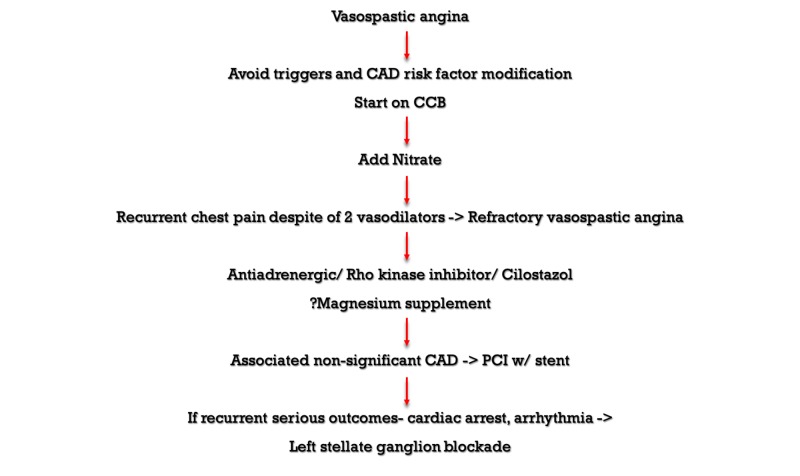
Vasospastic and refractory vasospastic angina treatment CAD - Coronary Artery Disease; CCB - Calcium Channel Blocker; PCI - Percutaneous Intervention

Management of vasospasm involves both medical and non-medical therapies (Figure 4). Management within daily living includes smoking cessation, alcohol cessation, as well as avoidance of triggers [9]. Aggressive CAD risk factor modification should also be pursued. Medication wise, CCBs are first line, with nitrates as second line. CCBs suppress calcium inflow into vascular smooth muscle cells, resulting in vasodilation. Nitrates are metabolized to nitric oxide, which subsequently activates guanylate cyclase, leading to increased cyclic guanosine monophosphate (cGMP) and, ultimately, relaxation of vascular smooth muscle. Nitric oxide also suppresses the activity of Rho-kinase, thereby also relaxing smooth muscle. For prevention, CCBs are effective in approximately 93% of patients with the efficacy of dihydropyridine being greater than non-dihydropyridine CCBs. The efficacy rate increases if two of these are combined to nearly 100% [9-12]. If nitrates are to be used for prevention, long-acting nitrates are preferred in combination therapy with CCB [9-12]. Statin therapy may also have an added benefit via endothelial nitric oxide or direct effects on the vascular smooth muscle [13].

Our patient presented with a lesser-known phenomenon, refractory VSA. Refractory VSA is when there is the continued presence of intermittent vasospasm despite being on a combination of two medications. Treatment for VSA is well documented, however, little data is available for refractory VSA. The literature frequently documents reports of several unconventional methods of treatment [14-15]. These treatment methods include the use of alpha-2-agonists, rho-kinase-inhibitors, statins, and magnesium [14-16].

Surgical interventions with sympathetic denervation like left-stellate-ganglion denervation have been explored and reported with mixed results for refractory VSA. Randomized control trials evaluating the efficacy of these unconventional management modalities are few to come by [17-19]. Further research and the sharing of the experiences of managing such perplexing refractory VSA cases should be encouraged to better understand and expand management.

## Conclusions

Vasospastic angina (VSA) occurs when there is spasm of the coronaries, transiently leading to constriction and eventual myocardial ischemia. VSA is treated typically with calcium-channel blockers and nitrates. However, there are times when the vasospasm is refractory to conventional medications. Refractory vasospastic angina is when angina continues despite being on a combination of two medications. When this occurs, unconventional treatment modalities may be employed for symptomatic relief. Diagnosis can be made via left heart catheterization with provocation testing with, typically, acetylcholine or ergonovine. Further research and sharing experiences of managing such perplexing refractory VSA cases should be encouraged to better understand and expand the management.

## References

[1] Teragawa H, Oshita C, Ueda T (2018). Coronary spasm: it's common, but it's still unsolved. World J Cardiol.

[2] Heberden W ( 1772). Some account of a disorder of the breast. Medical Transactions of the Royal College of Physicians.

[3] Prinzmetal M, Kennamer R, Merliss R, Wada T, Bor N (1959). Angina pectoris I. A variant form of angina pectoris. Am J Med.

[4] Beltrame JF, Crea F, Kaski JC (2015). The who, what, why, when, how and where of vasospastic angina. Circ J.

[5] Harris JR, Hale GM, Dasari TW, Schwier NC (2016). Pharmacotherapy of vasospastic angina. J Cardiovasc Pharmacol Ther.

[6] Ogawa H, Akasaka T, Hattori R (2014). Guidelines for diagnosis and treatment of patients with vasospastic angina (coronary spastic angina) (JCS 2013). Circ J.

[7] Takaoka K, Yoshimura M, Ogawa H (2000). Comparison of the risk factors for coronary artery spasm with those for organic stenosis in a Japanese population: role of cigarette smoking. Int J Cardiol.

[8] Amsterdam EA, Wenger NK, Brindis RG (2014). 2014 AHA/ACC Guideline for the Management of Patients with Non-ST-Elevation Acute Coronary Syndromes: a report of the American College of Cardiology/American Heart Association Task Force on practice guidelines. J Am Coll Cardiol.

[9] Beltrame JF, Crea F, Kaski JC (2017). International standardization of diagnostic criteria for vasospastic angina. Eur Heart J.

[10] Kimura E, Kishida H (1981). Treatment of variant angina with drugs: a survey of 11 cardiology institutes in Japan. Circulation.

[11] Io K, Minatoguchi S, Nishigaki K (2007). Effects of benidipine and some other calcium channel blockers on the prognosis of patients with vasospastic angina. Cohort study with evaluation of the ergonovine coronary spasm induction test. Arzneimittelforschung.

[12] Fukumoto Y, Yasuda S, Ito A, Shimokawa H (2008). Prognostic effects of benidipine in patients with vasospastic angina: comparison with diltiazem and amlodipine. J Cardiovasc Pharmacol.

[13] Yasue H, Mizuno Y, Harada E (2008). Effects of a 3-hydroxy-3-methylglutaryl coenzyme A reductase inhibitor, fluvastatin, on coronary spasm after withdrawal of calcium-channel blockers. J Am Coll Cardiol.

[14] Frenneaux M, Kaski JC, Brown M, Maseri A (1988). Refractory variant angina relieved by guanethidine and clonidine. Am J Cardiol.

[15] Shin ES, Lee JH, Yoo SY (2014). A randomised, multicentre, double blind, placebo controlled trial to evaluate the efficacy and safety of cilostazol in patients with vasospastic angina. Heart.

[16] Masumoto A, Mohri M, Shimokawa H, Urakami L, Usui M, Takeshita A (2002). Suppression of coronary artery spasm by the Rho-kinase inhibitor fasudil in patients with vasospastic angina. Circulation.

[17] Bertrand ME, Lablanche JM, Tilmant PY, Ducloux G, Warembourg H Jr, Soots G (1981). Complete denervation of the heart (autotransplantation) for treatment of severe, refractory coronary spasm. Arch Mal Coeur Vaiss.

[18] Clark DA, Quint RA, Mitchell RL, Angell WW (1977). Coronary artery spasm. medical management, surgical denervation, and autotransplantation. J Thorac Cardiovasc Surg.

[19] Abbate A, Hamza M, Cassano AD (2012). Sympathectomy as a treatment for refractory coronary artery spasm. Int J Cardiol.

